# Clinical value of SMI Combined with Low-Dose CT Scanning in differential diagnosis of Thyroid Lesions and Tumor Staging

**DOI:** 10.12669/pjms.37.5.4144

**Published:** 2021

**Authors:** Shao-wei Xue, Yu-kun Luo, Zi-yu Jiao, Lin Xu

**Affiliations:** 1 Shao-wei Xue, Department of Ultrasound Diagnosis, The First Medical Center of Chinese PLA General Hospital, Beijing 100853, P. R. China; 2 Yu-kun Luo, Department of Ultrasound Diagnosis, The First Medical Center of Chinese PLA General Hospital, Beijing 100853, P. R. China; 3 Zi-yu Jiao, Department of Ultrasound Diagnosis, The First Medical Center of Chinese PLA General Hospital, Beijing 100853, P. R. China; 4 Lin Xu, Department of Radiology, The First Medical Center of Chinese PLA General Hospital, Beijing 100853, P. R. China

**Keywords:** Superb Microvascular Imaging (SMI), low-dose CT scanning, thyroid nodules, differential diagnosis

## Abstract

**Objectives:**

To investigate the clinical value of Superb Microvascular Imaging (SMI) combined with low dose CT scanning in differential diagnosis of thyroid lesions and tumor staging.

**Methods:**

A total of 120 patients with thyroid nodules admitted to the Chinese PLA General Hospital from January 2017 to July 2020 were selected. Paired design was adopted in this study. SMI and SMI combined with low-dose CT scanning were respectively carried out to these patients. The results were judged by two senior imaging physicians and two senior sonographers respectively. And t-test, χ^2^ test, Pearson correlation coefficient check and other methods were adopted to comparatively analyze the above two methods and the pathological results after operation or puncture.

**Results:**

Compared with pathologic findings, the coincidence rate of SMI was 40%, and the coincidence rate of SMI combined with low dose CT scanning was 84%. The difference was significant (p=0.00); among the 120 thyroid nodule patients, 50 were diagnosed as malignant by pathological diagnosis, and 70 as benign; 27 malignant cases and 93 benign cases were detected by SMI; 48 malignant cases and 72 benign cases were detected by SMI combined with low dose CT scanning. The sensitivity and accuracy of the latter were significantly higher than those of the former, and the difference was statistically significant (p=0.00); the enhancement, edge sharpness and homogeneity of SMI increased with the increase of tumor malignancy, showing positive correlation property.

**Conclusion:**

SMI combined with low dose CT scanning has a higher diagnostic coincidence rate. Its sensitivity and accuracy are significantly superior. With the increase of tumor malignancy, the enhancement and unhomogeneity of SMI increase, and the edge is more blurred. That suggests: with the increase of tumor malignancy, neovascularization in the tumor is more obvious and more unevenly distributed; the increase of edge blur indicates more obvious tumor infiltration. The method has considerable clinical value for predicting the malignancy of tumors.

## INTRODUCTION

Thyroid nodules are one of the most common space-occupying lesions in neck. The incidence of adults is about 5%.^1^, and the incidence of females is higher than that of males. Although more than 90% of nodules are benign lesions, thyroid nodules are clinically important as they may represent approximately a thyroid cancer risk of 4.0% to 6.5%.^2^ Hence, careful assessment and exclusion must be carried out.^3^ At present, the diagnosis of thyroid nodules mainly depends on CT, ultrasonography and other imageological examinations.^4^ CT is highly sensitive to space-occupying lesions and can clearly show the boundary, internal morphological structure, size, cystic degeneration, calcification and so on of thyroid nodules, but CT is radioactive, and especially cephalic CT may do damage to the patients.^5^ Ultrasonic examination is non-invasive, simple and convenient, but it is difficult to clearly show the fine structure of the lesion. Superb Microvascular Imaging (SMI) is an advanced ultrasonic imaging technique. This technique, based on the fact that echo characteristics vary between human soft tissues, conducts intravenous injection of contrast agent to enhance the display of organs and lesions. Since the contrast agent microbubbles are small enough to pass microcirculation, SMI can display venules and arterioles, which is more conducive to judging the nature of pathological tissues.^6^ However, there are still limitations in the differential diagnosis of benign or malignant thyroid nodules. Especially when the nodules are of equal density, there is impact on the judgment results.^7^ The purpose of this study was to find a more advantageous method to determine the nature of thyroid nodules.

## METHODS

### Ethical Approval

The study was approved by the Institutional Ethics Committee of the first medical center of Chinese PLA General Hospital (No. S2019-178-02) in May 10, 2020, and written informed consent was obtained from all participants.

### Inclusion criteria:

1. Patients with thyroid nodules found on radiographic examination.
2. Patients with a definite pathological diagnosis (specimen sources: surgical specimens and puncture specimens)^8^.
3. Conscious patients without mental disorders.
4. Patients and their families are willing and able to cooperate to complete the study and have good treatment compliance.
5. Patients aged between 30 and 60 years and generally in good condition.

### Exclusion Criteria:

1. Patients who are not newly diagnosed.
2. Patients with non-thyroid tumors or other conditions that could affect the study results.
3. Patients with incomplete clinical or pathological data.
4. Patients suffering from mental illness or other cognitive impairment. All the subjects agreed to participate in the study and signed letters of informed consent.

A total of 120 patients with thyroid nodules admitted to the Chinese PLA General Hospital from January 2017 to July 2020 were selected and divided into malignant group and benign group after pathological examinations. Paired design was adopted in this study. There were 50 cases in malignant group, 19 males and 31 females aged 32~57, average age: 46.83±8.20, BMI: (22.15±1.24) kg/m2, maximum diameter of nodule: 4.21±0.76cm, course: 4.27±1.02 years. There were 70 cases in benign group, 28 males and 42 females aged 30~58, average age: 46.39±8.79, BMI: (23.36±4.57) kg/m2, maximum diameter of nodule: 2.73±0.54cm, course: 2.15±0.88 years. There is no significant difference between the two groups in general information (sex, age, BMI); there was significant difference (p=0.00) in disease condition- the diameters of nodules in malignant group are larger than those in benign group (Table-I). Pathological results: malignant group: 26 cases of papillary carcinoma, 12 cases of follicular carcinoma, 8 cases of medullary carcinoma and four cases of undifferentiated carcinoma; benign group: nodular goiter 36 cases, adenoma 24 cases, Hashimoto’s thyroiditis with nodule 5 cases, and other benign nodules 5 cases.

**Table-I:** Comparative analysis of general information between experimental group and control group (X̅ ±S)

Indicator	Malignant group (50 cases)	Benign group (70 cases)	t/χ^2^	P
Age (years old)*	46.83±8.20	46.39±8.79	0.28	0.78
Male (cases, %)	19(38%)	28(49%)	0.05	0.82
BMI (kg/m^2^)*	22.15±1.24	23.36±4.57	1.82	0.07
Nodule diameter (cm)Δ	4.21±0.76	2.73±0.54	12.47	0.00
Course (years)*	3.27±1.02	3.15±0.88	0.68	0.49

* P>0.05>Δ<0.05.

### Method of CT scan Low dose CT scanning

The patient lies supine, head leaned back and neck fully exposed. Philips Ingenuity 64-layer SCT is used, ranging from 3~5cm above the skull base to the level of thoracic entrance. Scanning parameters: 120 kV, 90mAs, thin layer scan, layer thickness and layer distance: 3-mm, pitch 0.87:1, tabletop speed 8.75 mm/r. Contrast agent for enhancement scan: iopromide, dose: 1.5 mL/kg. Delay time: 30s, 60s. Two senior physicians evaluated and recorded the CT values of the nodules and judged the modes of CT enhancement^9^: 1. circular enhancement: the periphery of the nodule is circular enhancement, and the density is higher than that of the inside; 2. homogeneous enhancement: the nodule is diffuse homogeneous enhancement; 3. nonhomogeneous enhancement: the nodule enhancement is inhomogeneously distributed; 4. punctate enhancement: only local parts show sparse punctate enhancement inside the nodule; 5. no enhancement: no obvious enhancement in the nodule.

### Method of SMI

The patient lies supine, head leaned back and neck fully exposed. Ultrasound: Philips color Doppler ultrasound, linear array probe: L5-12, frequency: 12MHz. First, routine ultrasonic scanning is performed, and the number, position, shape and size of nodules are recorded. Target nodules are selected for contrast-enhanced ultrasonography. The Bracco Sono Vuc contrast agent is taken and mixed with saline (5ml) to prepare microbubble suspension liquid. The liquid (2ml) is injected through the elbow vein. Rinse with 5.0mL saline after injection. Press the timer key during contrast injection. Observe the nodule perfusion for at least 60s continuously. The results are evaluated by two senior sonographers, and the images are recorded in time. Judgement criteria: (1) enhancement degree: the echo intensity of peak lesion enhancement is divided into weak enhancement, equal enhancement and strong enhancement compared with the surrounding thyroid parenchyma; (2) enhancement homogeneity: the homogeneity of peak lesion enhancement is divided into homogeneous enhancement and nonhomogeneous enhancement (including local non-enhancement).^10^

### Observation indicators

(1)The sensitivity, specificity and accuracy of SMI and SMI combined with low dose CT scanning in thyroid nodule differentiation (pathological results used as the diagnostic basis); (2) the coincidence rates between different examination methods and pathological results; (3) the correlations between different pathological types and SMI results.

### Statistical Method

All data are processed with SPSS 20.0 software, and measurement data are expressed as (±S). Intergroup data analysis is realized by t-test based on two groups of independent samples, and the rates are compared by χ^2^ test. The correlation is expressed by Pearson correlation coefficient, and P<0.05 is statistically significant.

## RESULTS

A comparative analysis of the two examination methods and the pathological results was conducted (Table-II). Among 50 malignant cases confirmed by pathological results, 20 cases were detected by SMI. The coincidence rate was 40%; 42 cases were detected by SMI combined with low dose CT scanning. The coincidence rate was 84%. The difference between the two groups was significant (p=0.00), suggesting a higher coincidence rate of SMI combined with low dose CT scanning.

**Table-II:** Comparative analysis of coincidence rates of different examination methods with pathological results(X̅ ±S)n=50

Method	Malignant	Pathological diagnosis	Coincidence rate*
SMI	20	50	40%
SMI combined with low dose CT scan	42	50	84%
χ^2^			20.54
p			0.00

* P<0.05.

Of 120 thyroid nodule cases, 50 were pathologically diagnosed as malignant and 70 as benign. Among them, 27 malignant cases and 93 benign cases were detected by SMI; 48 malignant cases and 72 benign cases were detected by SMI combined with low dose CT scanning. The sensitivity and accuracy of the latter were significantly higher than those of the former, and the difference was statistically significant (p=0.00). Table-III and Table-IV.

**Table-III:** Comparative analysis of thyroid nodule differentiation results between SMI and SMI combined with low dose CT scanning.

Pathological result	SMI			SMI combined with low dose CT scan		

	Malignant	Benign	Total	Malignant	Benign	Total
Malignant	20	30	50	42	8	50
Benign	7	63	70	6	64	70
Total	27	93	120	48	72	120

**Table-IV:** Analysis of sensitivity, specificity and accuracy of two groups.

Method	Sensitivity*	Specificity	Accuracy*
SMI	40.00%(20/50)	90.00%(63/70)	69.17%(83/120)
SMI combined with low dose CT scan	84.00%(42/50)	91.42%(64/70)	88.33%(106/120)
χ^2^	20.54	0.08	13.17
P	0.00	0.77	0,00

* P<0.05.

The correlation analysis between different pathological types and SMI results suggested: the enhancement, edge sharpness and homogeneity of SMI increased with the increase of tumor malignancy, showing positive correlation property (Table-V).

**Table-V:** Correlation analysis between pathological types and SMI results.

Type	Enhancement	Edge blur length	Nonhomogeneity
Papillary	0.32	0.35	0.23
Follicular	0.57	0.47	0.31
Medullary carcinoma	0.73	0.63	0.45
Undifferentiated carcinoma	0.81	0.74	0.47

## DISCUSSION

Thyroid nodules are masses of structural abnormality that occur in normal thyroid tissues due to certain causes. Thyroid nodules are the most common thyroid disease clinically. With the rising number of patients and the continuously improved examination methods, the detection rate of thyroid nodules is gradually increasing. Of all thyroid nodules, thyroid cancers account for less than 10%, and most nodules are benign. Benign nodules generally have regular morphology and a clear boundary with the surrounding normal thyroid gland. Malignant nodules are mostly irregular in morphology, and have unclear boundaries with normal thyroid and surrounding structures, with blurred edges and hard texture. At present, the most commonly used imageological examination for the diagnosis of thyroid nodules are CT and ultrasound. Although routine ultrasonic examination is simple, fast and non-invasive, and can provide information for the diagnosis of thyroid nodules, there is subjectivity in determining the boundary, echo and calcification conditions. Conventional CT does harm to head and neck tissues.

Tumor vessels are the most important characteristic of tumor tissues.^11^ Research shows that compared with benign tumors; malignant tumors have different microvascular architecture. It is characterized by distortion and angiectasis of tumor vessels and structural disorder.^12^ Therefore, Zhou et al.^13^ believe that Superb Microvascular Imaging (SMI) is a more accurate method to display tumor microvessels. Adabi et al.^14^ believe that vascular network can provide valuable information about tumor angiogenesis. SMI has been found effective in the differentiation of breast lesions, thyroid nodules and liver lesions in human body. SMI is more effective than CDI/PDI in detecting microvascular blood flow signals. For malignant nodules, SMI can more clearly describe the incompleteness of the peripheral microvascular system and the abnormalities of internal heterogeneous vascular system. Compared with conventional ultrasound, SMI can describe microvascular flow and vascular branches around and inside thyroid nodules in a more detailed and clear way. So it is a safe and low-cost scheme for distinguishing benign and malignant thyroid nodules.^15^

The main difference between malignant thyroid nodules and benign thyroid nodules is rapid angiogenesis. Blood vessels are usually richer and more disordered within malignant tissues. The study of Jiang et al.^16^ suggests: on contrast-enhanced ultrasound, most papillary thyroid carcinoma cases show heterogeneous low enhancement patterns, most nodular goiter patients show equal enhancement patterns, and adenoma patients show high enhancement patterns. Contrast-enhanced ultrasound is a practical and convenient method to identify malignant thyroid nodules. Hornung et al.^17^ believe that contrast-enhanced ultrasound is a highly sensitive method to detect microvascularization of thyroid cancer. Future studies should compare these findings with pathology of benign nodules to establish contrast-enhanced ultrasound as a standard diagnostic procedure for preoperative evaluation of suspicious thyroid nodules. The study of Carraro and Molinari suggests that. SMI has good consistency with biopsy results, so this method may be helpful for judging the degree of nodule differentiation in thyroid.^18^ Our study confirms: SMI and pathological types are correlated; the enhancement, edge blur length and inhomogeneity of SMI increase with the increase of tumor malignancy; thyroid nodules with high malignancy have abundant tumor vessels, which have great heteromorphism and infiltrate the surrounding tissues.

Nevertheless, contrast-enhanced ultrasound also has some defects. Mazzeo and his colleagues.^19^ believe that contrast-enhanced ultrasound may be helpful for some patients, while it may generate uncertain results in no enhancement patients. Ideally, another examination means is performed before a SMI examination is carried out, and then a comprehensive assessment result is offered. The findings of Li et al.^20^ show: the detection rates of conventional ultrasound and SMI decrease with the reduction of nodules, while CT is superior over ultrasound for small nodules. Traylor et al.^21^ believe that CT imaging is very important for preoperative evaluation of thyroid masses and thyroid cancers, while CT should be used as an important auxiliary means of SMI.^22^

The results of our study suggest that compared with pathologic findings, the coincidence rate of SMI was 40%, and the coincidence rate of SMI combined with low dose CT scanning was 84%. The difference was significant (p=0.00); among the 120 thyroid nodule patients, 50 were diagnosed as malignant by pathological diagnosis, and 70 as benign; 27 malignant cases and 93 benign cases were detected by SMI; 48 malignant cases and 72 benign cases were detected by SMI combined with low dose CT scanning. The sensitivity and accuracy of the latter were significantly higher than those of the former, and the difference was statistically significant (p=0.00). Therefore, the combination of SMI and low dose CT scanning is more helpful for making up for the judgment of equal density nodules in SMI so as to improve the accuracy of diagnosis.

### Limitations of this study

The combined method proposed by our study is still in the early stages of clinical application. Low-dose CT scanning and SMI are affected by many factors. In addition, the size of malignant sample is small. Further clinical exploration is necessary.

## CONCLUSION

SMI combined with low dose CT scanning has significantly higher coincidence rate, sensitivity and accuracy. With the increase of tumor malignancy, the enhancement and inhomogeneity of SMI increase, and the edge is increasingly blurred. That indicates that with the increase of tumor malignancy, neovascularization in tumor is more obvious and more unevenly distributed. And the increase of edge blur indicates more obvious tumor infiltration. The findings are of certain clinical value for predicting the malignancy of tumor.

### Authors’ Contributions:

**SWX** & **YKL:** Designed this study and prepared this manuscript, and are responsible and accountable for the accuracy or integrity of the work.

**LX;** & **ZYJ:** Collected and analyzed clinical data and significantly revised this manuscript.
